# Internet-based health education in China: a content analysis of websites

**DOI:** 10.1186/1472-6920-14-16

**Published:** 2014-01-27

**Authors:** Ying Peng, Xi Wu, Salla Atkins, Merrick Zwarentein, Ming Zhu, Xing Xin Zhan, Fan Zhang, Peng Ran, Wei Rong Yan

**Affiliations:** 1 Department of Epidemiology and Biostatistics, School of Public Health, Tongji Medical College, Huazhong University of Science and Technology, Wuhan, China; 2 Division of Global Health, Department of Public Health Science, Karolinska Institute, Stockholm, Sweden; 3 Knowledge Translation Unit, University of Cape Town Lung Institute, Cape Town, South Africa

## Abstract

**Background:**

The Internet is increasingly being applied in health education worldwide; however there is little knowledge of its use in Chinese higher education institutions. The present study provides the first review and highlights the deficiencies and required future advances in Chinese Internet-based health education.

**Methods:**

Two authors independently conducted a duplicate Internet search in order to identify information regarding Internet-based health education in China.

**Results:**

The findings showed that Internet-based education began in China in September 1998. Currently, only 16 of 150 (10.7%) health education institutions in China offer fee-based online undergraduate degree courses, awarding associates and/or bachelors degrees. Fifteen of the 16 institutions were located in the middle or on the eastern coast of China, where were more developed than other regions. Nursing was the most popular discipline in Internet-based health education, while some other disciplines, such as preventive medicine, were only offered at one university. Besides degree education, Chinese institutions also offered non-degree online training and free resources. The content was mainly presented in the form of PowerPoint slides or videos for self-learning. Very little online interactive mentoring was offered with any of the courses.

**Conclusions:**

There is considerable potential for the further development of Internet-based health education in China. These developments should include a focus on strengthening cooperation among higher education institutions in order to develop balanced online health curricula, and on enhancing distance education in low- and middle-income regions to meet extensive learning demands.

## Background

Medicine and health is closely related to human health and comprises a diverse collection of knowledge, skills, and practices, which should be updated frequently to meet the changing health demands. Well-trained healthcare professionals are crucial for the effective functioning of any health system [1], especially for a geographically large country like China, in which the healthcare demands vary greatly from region to region [2]. In the past, health education was primarily delivered through classroom lectures and printed materials, but these cannot be easily updated and disseminated; therefore, it is difficult to adapt them to rapid changes in healthcare demands and constant medical advances [3]. In addition, traditional training is rather inflexible in terms of time and space, requiring students to be present in a classroom for extended periods. This poses a large challenge to the provision of healthcare during training periods, since there is already a workforce shortage in healthcare settings, particularly in remote rural areas [4].

Internet-based education, which has been defined as using the Internet to deliver and access learning materials, interact with other learners and instructors, and obtain support during the learning process [5], is becoming increasingly popular in higher education. The effectiveness of Internet-based education methods has been demonstrated in meta-analyses and systematic reviews [6,7] - it is more effective than no teaching and it is equivalent to traditional teaching formats. It is argued that Internet-based training should be widely adopted in order to facilitate health education for equipping the healthcare workforce because it is very well suited for satisfying an extensive range of training needs and for transcending space and time zone [8]. Choules [9] declared that integrating information technologies into health education will relieve the problems mentioned above and will greatly benefit the community of teachers, learners and education institutions. Since there is little knowledge of the status of Internet-based health education in Chinese higher education institutions, we conducted this content analysis of websites to provide relevant information and discuss the future development of the field.

## Methods

### Information resources

Data on available websites with information on Internet-based health education in China were collected as follows: Two authors independently conducted Internet searches to identify websites on 1 September 2013, using the same search engines - Google and Baidu - and the same terms (‘e-learning’; ‘Internet-based’; ‘web-based’; ‘online course’; ‘health’; ‘medical’; ‘China’; ‘Chinese’; and ‘higher education institutions’). We conducted these searches both in English and in Chinese. Then the websites were reviewed by one author and another author reviewed the same sites to retrieve information conforming to criteria outlined below. If there was any conflict or inconsistency between the two authors, a discussion was organized to resolve the conflict and reach a consensus.

### Data selection

Of the nigh-endless list of websites retrieved, 500 of the most relevant sites (selected by ranking according to their relevancy to the search terms) and specific sites that were considered interesting/helpful were evaluated by each author for their relevancy to the study purpose. The following criteria were applied to determine whether the information was relevant to this study and its aims: (1) had highly objective data (i.e. presented by higher education institutions or organizations); (2) had freely available content; (3) had regularly updated sites (i.e., evaluated through the ‘last updated’ date on the website within a one-year interval of the time of review); and (4) contained information relevant to Internet-based health education in China.

We reviewed the online health education programmes as to what disciplines they offered, whether they offered a formal degree after students completing courses or if they just provided online open courses for self-learning without awarding a degree. We also reviewed how they enrolled students and what web-based support platform they used. In the case of a disagreement between the two authors, they discussed with the third author, and the results were attained by consensus. The review process was completed on 15 October 2013.

## Results

In total, 21 websites were considered to be of merit according to the consensus of the authors. The websites searching for relevant information are listed in Additional file 1: Table S1, and Table 1 summarizes the selected website information on Internet-based degree education in Chinese health education institutions.

**Table 1:** Health disciplines in Internet-based degree education in China

**Name of University**	**Internet-based education platform**	**Offered disciplines**	**Degree**	Duration*
**1. Peking University**				
	Self-developed "Teaching Operation Support System, TOSS" platform	Nursing	Associate/Bachelor	2.5-6 years
		Pharmacy	Associate/Bachelor	2.5-6 years
		Applied psychology	Bachelor	2.5-6 years
**2. Beijing University of Chinese Medicine**				
	Blackboard (BB) platform, which is a famous online course management system worldwide	Herbology	Associate/Bachelor	2.5-5 years
		Acupuncture and moxibustion	Associate/Bachelor	2.5-5 years
		Nursing	Associate/Bachelor	2.5-5 years
		Medicine and health management	Associate/Bachelor	2.5-5 years
**3. China Medical University**				
	Chinese digital College (nclass) supported platform	Nursing	Associate/Bachelor	3 years
		Pharmacy	Associate/Bachelor	3 years
**4. Jilin University**				
	Self-developed multimedia education platform	Nursing	Associate/Bachelor	2.5-5 years
		Pharmacy	Bachelor	2.5-5 years
**5. Shanghai Jiaotong University**				
	Self-developed "Shanghai Jiaotong University-Open Mobile Real (SJTU-OMR) PPClass online course system" platform	Nursing	Associate/Bachelor	2.5-5 years
		Pharmacy	Associate/Bachelor	2.5-5 years
		Medicine and health management	Associate/Bachelor	2.5-5 years
		Medical laboratory technology	Associate/Bachelor	2.5-5 years
		Clinical engineering technology	Associate	2.5-5 years
		Biomedical engineering	Bachelor	2.5-5 years
**6. Zhejiang University**				
	Not clear	Nursing	Associate/Bachelor	2.5-5 years
		Pharmacy	Associate/Bachelor	2.5-5 years
**7. Southeast University**				
	Chinese digital College (nclass) supported platform	Nursing	Associate/Bachelor	2.5-5 years
**8. Shandong University**				
	Not clear	Nursing	Associate/Bachelor	2.5-5 years
		Pharmacy	Associate/Bachelor	2.5-5 years
		Medicine and health management	Associate/Bachelor	2.5-5 years
		Labor and social security	Associate/Bachelor	2.5-5 years
		Social work (family planning)	Associate	2.5-5 years
**9. Zhengzhou University**				
	Self-developed "Zhengzhou University Distance Learning Support (ZZU-DLS)" platform	Nursing	Associate/Bachelor	3-6 years
		Pharmacy	Associate/Bachelor	2.5-5 years
		Basic medicine	Bachelor	3-6 years
**10. Huazhong University of Science & Technology**				
	Self-developed real-time video conference teaching system and non-real-time online learning system	Nursing	Associate/Bachelor	2.5-5 years
		Pharmacy	Associate/Bachelor	2.5-5 years
		Medicine and health management	Bachelor	2.5-5 years
**11. Wuhan University**				
	Blackboard (BB) platform, which is a famous online course management system worldwide	Nursing	Associate/Bachelor	2.5-5 years
		Preventive medicine	Bachelor	2.5-5 years
**12. Centralsouth University**				
	Blackboard (BB) platform, which is a famous online course management system worldwide	Nursing	Associate/Bachelor	2.5-5 years
		Pharmacy	Bachelor	2.5-5 years
**13. Sun Yat-Sen University**				
	Blackboard (BB) platform, which is a famous online course management system worldwide	Nursing	Associate/Bachelor	3-5 years
		Medicine and health management	Bachelor	2.5-5 years
**14. Sichuan University**				
	Not clear	Nursing	Associate/Bachelor	2.5-5 years
		Pharmacy	Bachelor	2.5-5 years
		Food hygiene inspection	Associate	2.5-5 years
**15. Xi’an Jiaotong University**				
	Self-developed "YOTTA system" platform	Nursing	Associate/Bachelor	2.5-6 years
		Pharmacy	Associate/Bachelor	2.5-6 years
**16. Lanzhou University**				
	Self-developed "Internet-based education and management system" platform	Nursing	Associate/Bachelor	2.5-7 years
		Medicine and health management	Associate/Bachelor	2.5-7 years

*Flexible academic years within which required credits should be completed.

### Colleges or universities in China conducting web-based education

**Figure 1:**
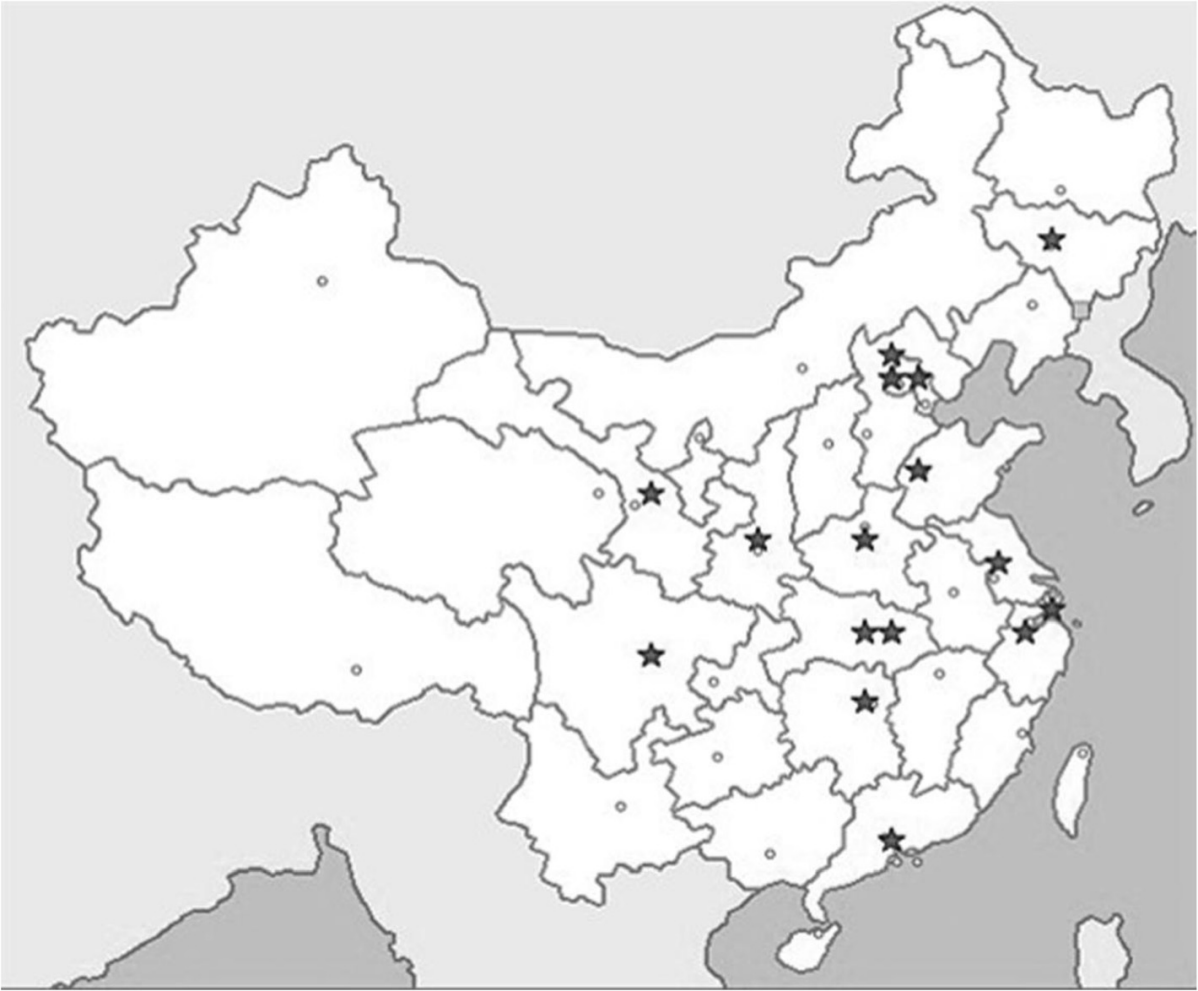
Locations (indicated with ‘★’) of the 16 institutions offering health-related disciplines via Internet-based degree education in China.

Tsinghua and nine other universities in China initiated the ‘China Education and Research Network (CERNET)’ at the end of 1994. Its network infrastructure mainly served universities, institutes, colleges, and schools all over China, laying the foundation for Internet-based education in Chinese higher education. In September 1998, the Ministry of Education officially accredited Tsinghua University, Beijing University of Post & Telecommunications, Zhejiang University, and Hunan University as the pilot sites for Internet-based education in China. Since then, there have been a growing number of attempts to use the Internet to deliver education and training in China. Currently, there were about 150 Chinese higher education institutions providing health education, 16 of which (10.7%) have gained accreditation to provide degree education over the Internet. The locations of these 16 institutions are demonstrated in Figure 1. Fifteen of the 16 institutions were located in the centre or on the eastern coast of China, which are the more developed regions.

### Fee-based courses and degree/certificate programs

**Figure 2:**
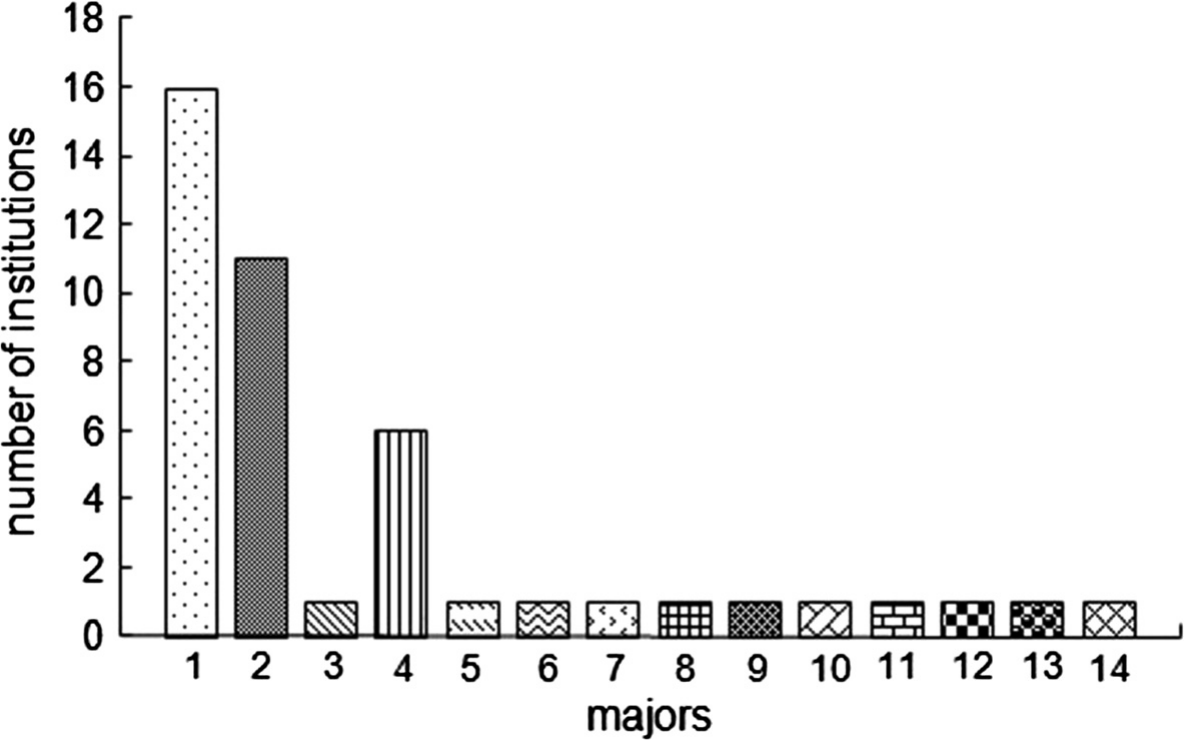
**Number of Chinese institutions for each health related discipline recently offered through Internet-based degree education.** Legend text:1 = Nursing; 2 = Pharmacy; 3 = Applied psychology; 4 = Medicine and health management; 5 = Herbology; 6 = Acupuncture and moxibustion; 7 = Medical laboratory technology; 8 = Clinical engineering technology; 9 = Biomedical engineering; 10 = Labor and social security; 11 = Social work (family planning); 12 = Basic medicine; 13 = Preventive medicine; 14 = Food hygiene inspection.

These 16 institutions have only awarded undergraduate (associate’s^a^ and/or bachelor’s) degrees so far. Nursing was by far the most popular discipline offered by all the institutions, followed by pharmacy, which is taught in 11 institutions. By comparison, some disciplines were available only at one university, such as ‘applied psychology’ at Peking University, ‘preventive medicine’ at Wuhan University, and ‘social work (family planning)’ at Shandong University. The numbers of Chinese institutions offering degrees through the Internet for each health-related discipline can be seen in Figure 2.

In order to obtain an Internet-based education degree, a student must complete 75 to 160 online course credits within 2.5 to 6 academic years, depending on the discipline and university. Only after obtaining the required credits can an enrolled student be awarded the degree. In addition to degree education, Peking University and Shandong University provide a one- to three-year postgraduate training courses for clinical and basic medicine through the Internet, awarding certificates and credits but no degree.

### Recruitment and admission

**Figure 3:**
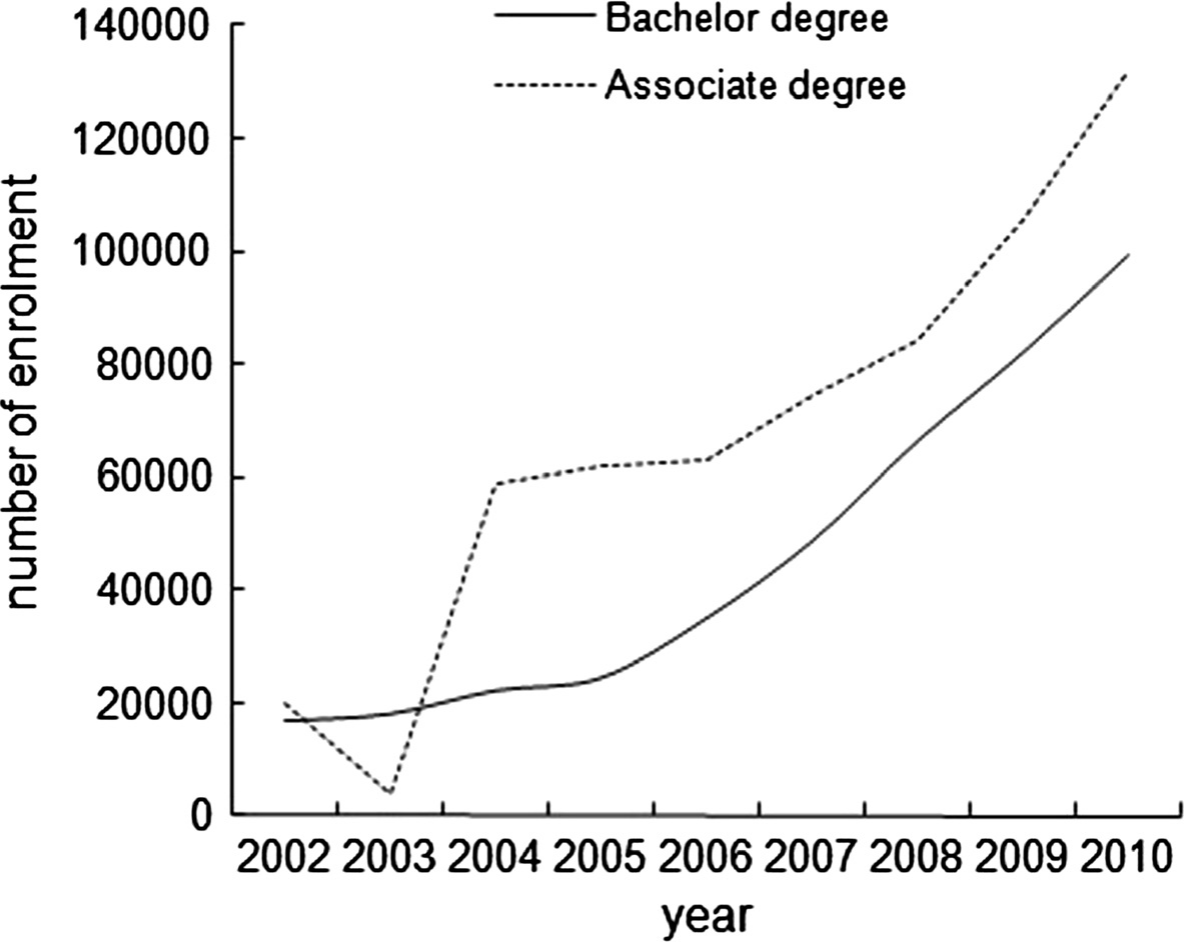
The trend of student enrollments in Internet-based health education from 2002 to 2010 in China.

Previous academic achievement and entrance examinations were required in order to be qualified for admission at all schools. Enrolled students were mainly adults, either with or without employment, and the number of students enrolled in Internet-based education in China has been rising since 1998. The latest national statistics from the Ministry of Education demonstrated that more than 1.1 million students had gained degrees through online education by 2010, and 3.96% of those were in health education. Figure 3 shows the trend of student enrolment in Internet-based health education from 2002 to 2010. It showed a sustained growth in the number of students taking part in bachelor’s degrees programmes. The number of students pursuing associate degrees temporarily declined in 2003; however, the number increased again in 2004, whereupon it surpassed the number of students in bachelor’s degree programmes.

### Platform and mode of delivery in Internet-based education

The platforms for most of these colleges/universities (7/16) were self-developed. These included the ‘Teaching Operation Support System (TOSS)’ at Peking University and ‘Yotta Network Platform’ at Xi’an Jiaotong University. Four institutions have introduced Blackboard (BB), which is a famous worldwide online course-management system. Detailed information about this platform’s usage can be seen in Table 1.

The primary learning mode in Internet-based education was found to be online self-learning. Teaching materials were presented in various forms, including PowerPoint slides (PPT), Word files, and videos. Students were required to study the courses in a given period, and then were required to complete online self-assessment quizzes and assignments. They also had the option of interacting with fellow students and instructors asynchronously and synchronously via e-mail, Bulletin Board Systems (BBS), and online chatting software. At the end of every semester, one week of face-to-face tutoring and online final examination was conducted.

### Open learning resources developed by Chinese higher education

Higher education institutions in China have also developed some free Internet-based learning resources for undergraduate students in recent years. One of these was the National Exquisite Course Project. This project was launched by the Ministry of Education in April 2003 to promote the development of high-quality online courses in Chinese higher education institutions. The courses developed in this project were put on the website of "National Exquisite Courses" (see Additional file 1: Table S1). Another source of free online courses was the Chinese University Consortium for Open Resources (CUCO) programme, which was established in December 2011 in order to foster university cooperation in sharing online courses and lectures by making them available to all students through the Internet (see Additional file 1: Table S1). It was coordinated by Peking University and encompassed 103 universities. A repository of courses was established on the CUCO network website. Compared to engineering and science, courses related to health in these Internet-based learning programmes were scarce and mainly focused on basic and clinical medicine, while courses focusing on preventive medicine and public health were very few. Currently, only 20 courses on the CUCO network focused on nursing, pharmacy, and Chinese medicine, and there were zero visits to some of these courses, such as geriatric nursing and drug therapeutics.

## Discussion

Chinese Internet-based education is in its infancy, but it has seen increasing enrolment since 1998. Our search findings showed that, despite the drawbacks, there is considerable potential for developing Internet-based health education in China.

In this information technology era, increasingly more people are eager to receive continuing education. However, it has been reported that, among 18- to 24-year-olds in China, only 2.5% have had the opportunity to participate in continuing education via traditional training models [10]. With the development and popularization of the Internet in recent years, Internet-based distance education has become widespread. It provides virtual learning opportunities for people without imposing time and geographical constraints. The integration of information technologies into health education in China could meet the nation’s extensive and varied demand for education, thereby supporting healthcare systems all over China and worldwide.

The 16 universities offering Internet-based education have so far provided only fee-based undergraduate degree education focusing on common disciplines. Greater capacity should be built into existing institutions so that they can provide Internet-based health education at not only an undergraduate level but also a postgraduate level in order to meet the growing demands for higher education, as was proposed in Wei’s master thesis [11].

The courses should also be optimized to meet practical health demands. In addition to clinical and nursing courses, which are related to the hospitalization and rehabilitation of human diseases, courses regarding prevention and promotion of public health, such as epidemiology, disaster health, disease surveillance and control, social determinants of health, and community health management programs, should be offered. These courses are currently lacking in the online education system in China while are definitely needed in practice.

Policy makers and researchers worldwide are gradually realizing the significance of improving Internet-based education programmes in the public health fields. For example, an EU supported FP7 project, the Asian Regional Capacity Development for Research on Social Determinants of Health (ARCADE RSDH), was launched in December 2011. This programme focused on strengthening institutions and enabling them to provide more Internet-based doctoral and post-doctoral training in the area of social determinants of health [12]. Three Chinese universities are currently taking part in this program – Huazhong University of Science and Technology, Beijing Normal University, and Zhejiang University. The project is expected to enrich Internet-based health education in China.

Additionally, the Internet is being used to deliver free learning material worldwide, with examples like the Harvard-MIT collaboration and the US Hewlett projects [13,14]. Similarly, in China, the National Exquisite Course Project and the CUCO programme aim to develop free online learning materials. However, courses on health topics provided by these resources were rare at the time of this search. Furthermore, the learning materials available are mainly presented in the form of PPT, Word files, or videos for self-learning. The provision of static courses online is not enough for a good learning experience because it lacks key interactions among learners. Intellecturally stimulating exchange of ideas has been cited as one of the key components of effective learning [15]. Experts suggest that distance education programmes are only successful if there is some provision for two-way communication in the educational process, with guidance, counseling, and timely feedback on assignments [16]. With the development of information technology, plenty of different media, such as CDs, DVDs, BBS, e-mail, and Internet-based video conferencing programs have been applied in Internet-based education to realize synchronous and/or asynchronous teaching–learning interactions. Taking advantage of modern communication technology allows for greater flexibility in implementing online education [17].

Online courses are convenient for universities and educational institutions to deliver. However, it is noteworthy that financial and technical support and quality assurance are essential to conducting successful Internet-based education programmes [18]. The findings from the searches revealed that the platforms for supporting Internet-based education in most colleges/universities were self-developed, which could be time-consuming, technically challenging and not cost-effective. Actually, there are some powerful existing Internet-based education supporting platforms, like BB and Moodle, which have been used worldwide [19,20]. However, only four out of these 16 institutions have introduced BB, and not one has introduced Moodle, to support Internet-based health education. It is advisable to take advantage of such powerful platforms. Besides, there have been a number of studies assessing the effectiveness of Internet-based health education [5,6] and guidelines have been developed on how online courses should be executed and evaluated [7]. However, nearly all of the research on distance learning and e-mentoring has come from the West. Very few studies have been carried out within a Chinese context. It has been suggested that in addition to the culture, the thinking patterns and learning habits of students, the traditional education model, and pedagogical theories differ between China and the West [21,22]. In view of this, there is a pressing need to perform studies in China to explore and guide the optimal development of Internet-based education to meet the specific demands of Chinese learners.

This study has several limitations. All of the information was extracted from websites. Though it unlikely, there might be other Internet-based health education programmes and free online resources as yet unidentified in our search. Another concern was the reliability of the information available on these websites. This is an eternal question when handling Internet-based information. In this case, however, there is an added measure of security because all of the websites selected for review were presented by authoritative higher education institutions and organizations. Furthermore, the representativeness of Internet-based higher education platforms in China could be somewhat attenuated because some of the facilities we searched did not give access to all the relevant information. In addition, as the online education platforms of each university are not open to the public, we could not log in to the platforms to further explore their management and operation processes. Given the fluidity of the web, the time-efficiency of the findings in the present research must also be taken into consideration. In the next year, it is likely that there will be considerable progress and updated information on Internet-based health education in China, and this will necessitate further research and description.

## Conclusions

In summary, Internet-based education seems to be gaining acceptance among students, as enrolment is increasing in China. However, the extent and strength of the current online health education is limited. There is a huge potential for its further development and improvement. The results of the present study provide background information for relevant stakeholders on how to improve online education. Further research that is specific to China is needed on the following topics: evaluating the quality of existing online learning resources; estimating the effectiveness of current Internet-based health education delivery; and developing guidelines for designing and assessing online health related courses and their implementation.

## Endnotes

^a^Associate degree: an undergraduate academic degree upon completion of courses of study, generally lasting 2-5years, for a specific occupation or a jump start to a bachelor’s degree.

## Competing interests

The authors declare that they have no competing interests.

## Authors’ contributions

YP contributed to the acquisition, analysis and interpretation of data, and drafted the article. XW contributed to the acquisition, analysis and interpretation of data. SA and MZw wrote the original protocol, were co-applicants on the grant application. MZh, XXZ, FZ and RP contributed to the acquisition of data. WRY wrote the original protocol, was co-applicant on the grant application and contributed to the conception and design of the study. All authors contributed to the revision of the paper and approved the final manuscript for submission.

## Pre-publication history

The pre-publication history for this paper can be accessed here:

http://www.biomedcentral.com/1472-6920/14/16/prepub

## Supplementary Material

Additional file 1: Table S1 Websites searched for relevant information. Click here for file
