# Supplementary material for: Internet-based health education in China: a content analysis of websites
Source: BMC Med Educ. 2014 Jan 27;14:16. doi: 10.1186/1472-6920-14-16 (PMC3910239; doi:10.1186/1472-6920-14-16)
Supplement: Additional file 1: Table S1 — Websites searched for relevant information. [file 1472-6920-14-16-S1.doc]

**Additional file 1: Table S1**

Websites searched for relevant information.

| **Name of the institute** | **website** |
| --- | --- |
| **Higher education institutions** |  |
| Peking University | <http://www.pkubytime.com.cn/> |
| Beijing University of Chinese Medicine | http://www.ibucm.com/ |
| China Medical University | <http://202.118.42.3/default.asp> |
| Jilin University | http://dec.jlu.edu.cn/ |
| Shanghai Jiaotong University | <http://www.mechina.org/index.php> |
| Zhejiang University | http://xywh.scezju.com/ |
| Southeast University | <http://netu.js.edu.cn/> |
| Shandong University | <http://sv6.wljy.sdu.edu.cn/ce/> |
| Zhengzhou University | <http://dls.zzu.edu.cn/> |
| Huazhong University of Science and Technology | <http://www.hust-snde.com/hust/newHust/index.jsp> |
| Wuhan University | <http://cce.whu.edu.cn/index.php> |
| Centralsouth University | <http://www.cnecsu.cn/index.html> |
| Sun Yat-Sen University | <http://www.ne.sysu.org.cn/> |
| Sichuan University | <http://www.scude.cc/> |
| Xi'an Jiaotong University | <http://www.dlc.xjtu.edu.cn/> |
| Lanzhou University | <http://www.dec.lzu.cn/> |
| **National institutes** |  |
| China Distance and Continue Education (CDCE) | <http://www.cdce.cn/> |
| China Education and Research Network | <http://www.edu.cn/> |
| Ministry of Education of the People’s Republic of China | http://www.moe.edu.cn/ |
| National Exquisite Courses | <http://www.jingpinke.com/> |
| Chinese University consortium for open resources | <http://istudy.pkudl.cn/Index.aspx> |
